# Depression and Nystagmus as the Rare Masquerading Presentations of Pineal Germinoma

**DOI:** 10.7759/cureus.42497

**Published:** 2023-07-26

**Authors:** Kah-Hie Wong, Teck-Chee Cheng, Suria Hayati Md Pauzi, Wan Haslina Wan Abdul Halim, Norshamsiah Md Din

**Affiliations:** 1 Department of Ophthalmology, Universiti Kebangsaan Malaysia Medical Centre, Kuala Lumpur, MYS; 2 Department of Pathology, Universiti Kebangsaan Malaysia Medical Centre, Kuala Lumpur, MYS

**Keywords:** pineal germinoma, parinaud’s syndrome, behavioral changes, depression, convergence-retraction nystagmus

## Abstract

Convergence-retraction nystagmus alongside behavioral changes can be rare manifestations of a potentially life-threatening midbrain lesion. After experiencing headaches for three months, a 13-year-old boy was diagnosed with depression due to exhibiting reduced speech, hypersomnia, and psychomotor slowing for three weeks. It was preceded by headache for three months. Examination revealed visual acuity of 6/6 bilaterally, convergence-retraction nystagmus worst on upgaze, limited bilateral ocular motility in upgaze, and light-near dissociation on pupil examination, all of which point towards Parinaud’s syndrome. However, there was no lid retraction to suggest Collier’s sign. Fundus examination revealed papilledema. Magnetic resonance imaging showed a large pineal mass extending to both thalami, dilated ventricles due to obstructive hydrocephalus, and cerebral edema. An urgent external ventricular drain was inserted, and biopsy revealed pineal gland germinoma. Chemotherapy and radiotherapy resulted in adequate tumor shrinkage. This case report highlights that subacute behavioral changes may mask a potentially life-threatening intracranial tumor, especially when associated with abnormal eye movement.

## Introduction

Brain tumors can cause behavioral changes. Frontal lobes are responsible for mediating various aspects of social functioning [1]. Therefore, frontal tumors can cause significantly higher levels of apathy, disinhibition, and executive dysfunction than non-frontal brain tumors [2]. When behavioral changes are associated with systemic signs, organic intracranial pathology should be suspected, instead of being assumed as psychiatric pathology without further workup. The possible systemic signs include anosmia, limb weakness, and expressive dysphasia.

On the other hand, behavioral and speech changes are rare presentations of pineal tumors and could mimic depression and other psychiatric disorders [3]. Instead of behavioral changes, pineal masses are more likely to present with non-specific neurological symptoms or signs (e.g., headache and seizure), signs from compression of local structures, or endocrine disturbances [4]. Direct compression of the midbrain results in cerebellar, corticospinal, or sensory disturbances [5]. Compression of the aqueduct of Sylvius leads to obstructive hydrocephalus and symptoms of raised intracranial pressure [4]. Compression of the superior colliculi results in Parinaud’s syndrome [4]. The commonest endocrine dysfunction is precocious puberty, less commonly hypogonadism and diabetes insipidus [4].

Subtle nystagmus is a sign that might be easily missed during examination, especially for non-ophthalmically trained or non-neurologically trained personnel. Various types of nystagmus point towards different underlying central or peripheral etiologies. Convergence-retraction nystagmus is characterized by quick rhythmic globe convergence and retraction, particularly during upward gaze. Along with upgaze palsy, light near dissociation, and bilateral lid retraction, the constellation of these four signs is known as Parinaud’s syndrome. It is a rare manifestation and has a high localizing value of life-threatening midbrain lesions such as pineal tumors.

Accurate and timely identification of systemic signs is crucial in ensuring that potentially life-threatening intracranial pathology is not missed. We aim to describe an uncommon presentation of behavioral changes and nystagmus in a potentially life-threatening pineal lesion.

This case was partly presented as a poster at the 11th Malaysian Society of Ophthalmology Annual Scientific Meeting on March 27-28, 2021.

## Case presentation

Following three months of headaches, a 13-year-old boy was diagnosed with depression by his psychiatrist due to experiencing reduced speech, hypersomnia, and psychomotor delay for the past three weeks. He was treated with escitalopram, risperidone, and lorazepam. There was no neuroimaging done prior to the diagnosis of depression. In addition, he was noted to have abnormal rhythmic eye movements for five weeks by his mother. A week later, he developed left hemiplegia and was brought to the emergency department. His nystagmus was noticed by the emergency physician, who referred him to the ophthalmology team. He had no pre-existing systemic medical disorders and no significant family history of malignancy.

**Figure 1:**
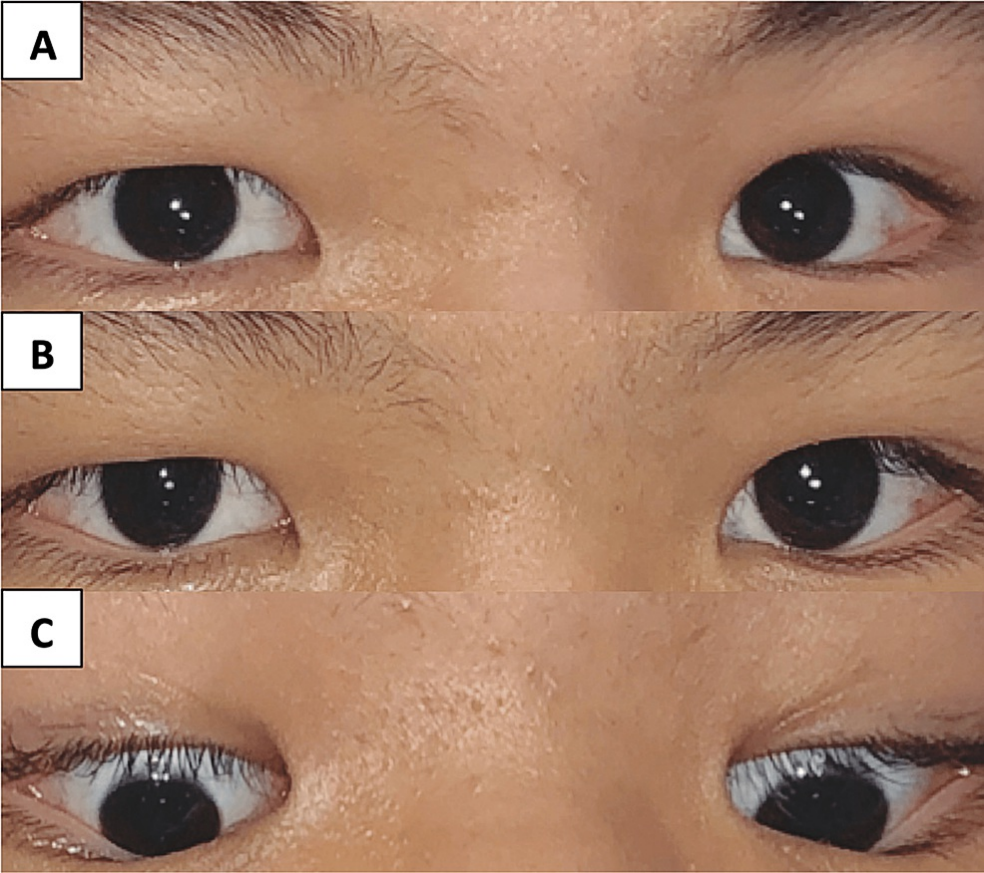
These photos show the ocular motility in upgaze (Figure 1A), primary gaze (Figure 1B), and downgaze (Figure 1C). There was limitation in bilateral upgaze (Figure 1A). There was convergence-retraction nystagmus in upgaze, primary gaze, and downgaze. The convergence-retraction nystagmus was the worst in upgaze attempt than other gazes.

On ocular examination, his visual acuity was 6/6 bilaterally. Convergence-retraction nystagmus was noted at upgaze where it was the worst, and also at primary gaze and downgaze (Figure 1). Ocular motility was limited in upgaze for both eyes (Figure 1). No Collier’s sign was noted. Pupil examination showed light-near dissociation without relative afferent pupillary defect. Fundus examination revealed bilateral hyperemic and swollen optic discs, which was compatible with papilledema. Other optic nerve function tests including direct pupil reflex, confrontational visual field, color vision, red saturation, and light brightness sensitivity were unremarkable. Neurological examination demonstrated weakness of the left upper and lower limbs with muscle power graded 3 out of 5. Otherwise, the cranial nerves, muscle tone, sensation, and reflexes were normal bilaterally.

**Figure 2:**
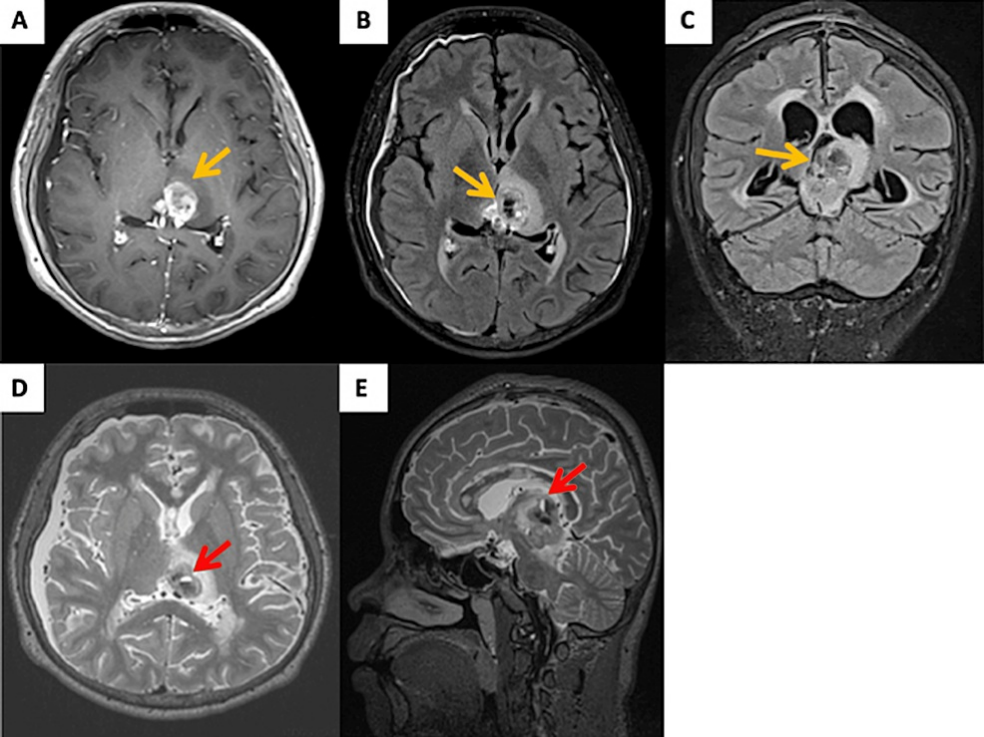
MRI images show a large pineal mass in (A) T1-contrasted axial cut (orange arrow), (B) T2-FLAIR axial cut (orange arrow), (C) T2-FLAIR coronal cut (orange arrow), (D) T2-contrasted axial cut with fluid level (red arrow) in tumor, and (E) T2 sagittal cut with fluid level (red arrow) in tumor. FLAIR, fluid-attenuated inversion recovery; MRI, magnetic resonance imaging

Magnetic resonance imaging (MRI) of the brain revealed a large lobulated cystic mass within the pineal gland, measuring 3.0 x 2.8 x 3.2 cm, with heterogenous hyperintensity on T1-weighted images (Figure 2). Within the tumor, there were fluid-fluid levels suggestive of a hemorrhagic component. The mass extended superiorly to both thalami, inferiorly to the midbrain involving the left cerebral peduncle, and anteriorly to the cerebral aqueduct, resulting in dilated lateral ventricles and third ventricles. The MRI findings suggested obstructive hydrocephalus with possible seepage. MRI of the spine showed no spinal cord metastasis.

An external ventricular drain was inserted urgently by the neurosurgical team to relieve the raised intracranial pressure and obtain cerebrospinal fluid (CSF) samples for further investigation of the pineal tumor.

Hormonal workup revealed normal serum beta-human chorionic gonadotropin (ß-HCG) levels and alpha-fetoprotein levels, suggesting low likelihood of a non-germinomatous germ cell tumor. Other hormones (testosterone, growth hormone, thyroid-stimulating hormone, free thyroxine-4, random cortisol, prolactin, estradiol, follicle-stimulating hormone, luteinizing hormone), serum osmolality, urine osmolality, and urine sodium were normal, suggesting that the tumor was non-secretory and did not result in precocious puberty or diabetes insipidus. This normal baseline hormonal profile was also important for monitoring of low hormonal secretion after radiotherapy.

Lumbar puncture revealed high opening pressure, but there was no tumor seeding or signs of infection in the CSF analysis. Other CSF biochemical analyses showed normal alpha-fetoprotein, raised ß-HCG (40 mIU/mL), and raised alkaline phosphate (314 U/L), which were expected of a germinoma.

**Figure 3:**
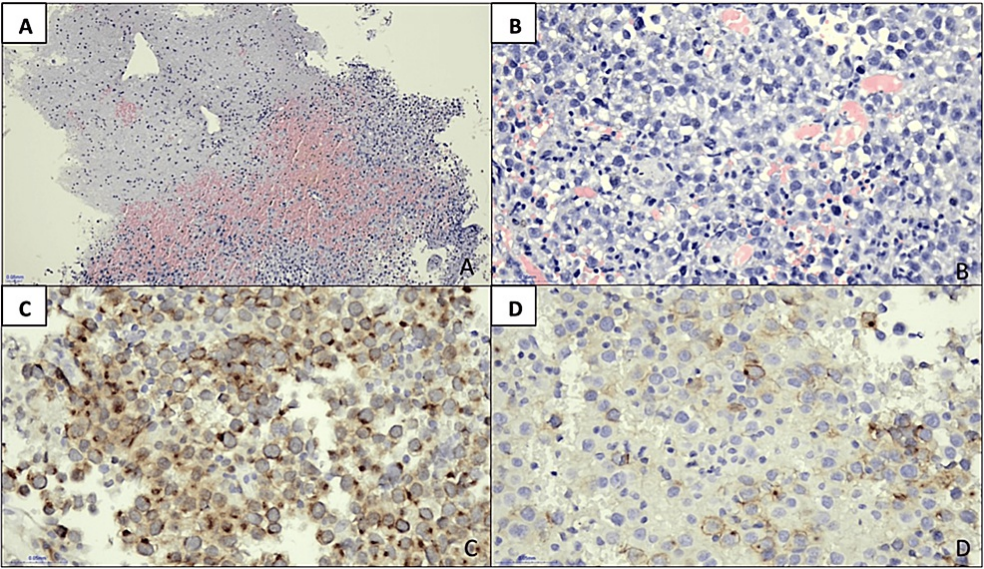
Microscopic images of the pineal tumor. (A) Fragments of normal brain (upper left) with adjacent highly cellular tumor fragments (lower right) (H&E, 10x). (B) The tumor cells exhibit round nuclei and prominent nucleoli with lymphocytes seen in between tumor cells (H&E, 40x). The tumor cells are immunopositive to (C) CD117 (40x) and (D) PLAP (40x). H&E, hematoxylin and eosin stain; PLAP, placental alkaline phosphatase

Image-guided stereotactic biopsy revealed a pineal gland germinoma. Histopathologic examination (Figure 3) exhibited (1) mild pleomorphism with moderate to large round hyperchromatic nuclei, prominent nucleoli, and moderate cytoplasm; (2) occasional mitotic figures; (3) mild lymphocytic infiltration in the surrounding parenchyma; (4) neoplastic cells, which stained positive with CD 117 diffusely and placental-like alkaline phosphatase (PLAP) patchily at the membranes and cytoplasm; (5) neoplastic cells unstained with synaptophysin and chromogranin.

The mainstay of his treatment was chemotherapy and radiotherapy. No surgical resection was performed. Technetium-99m diethylene triamine penta-acetic acid dynamic renal scintigraphy scan showed overall good kidney function. Therefore, the patient underwent chemotherapy and radiotherapy according to the current treatment guideline [6,7]. He underwent four cycles of chemotherapy with cycles 1 and 3 using etoposide and carboplatin and cycles 2 and 4 using etoposide and ifosfamide. Subsequently, he underwent radiotherapy of 24 Gy in 15 fractions for whole ventricles and 16 Gy in 10 fractions of boost radiation to the tumor bed. The tumor responded well to the treatment and shrunk in size from 3.0 x 2.8 x 3.2 cm to 1.7 x 0.7 x 0.6 cm (anteroposterior x width x craniocaudal dimension respectively) at the end of treatment. He was followed up by the pediatric oncology team with MRI for surveillance, initially 4 monthly in the first year and then subsequently yearly. So far, he has achieved 30-month survival period free of tumor relapse and metastasis. His behavioral changes, i.e., reduction in speech, psychomotor slowing, and hypersomnia resolved halfway through his chemotherapy. However, when he was last seen by the ophthalmology team two years after diagnosis of pineal germinoma, his convergence-retraction nystagmus, upgaze palsy, and light-near dissociation persisted.

## Discussion

Pineal germinomas are a type of pinealomas, which can be benign or malignant. Pinealomas attribute to less than 1% of all primary intracranial tumors [8,9]. Germ cell tumors represent 50% of tumors found at the pineal region, with the majority being pure germinomas [8,9]. The median age of diagnosis for central nervous system germ cell tumors is 10-12 years [8]. Males are predominantly affected, with a male-to-female ratio of up to 3:1 for pineal tumors, and this ratio triples in pineal germ cell tumors with a ratio of 11.8:1 [8,9]. Nonetheless, germ cell tumors have the best overall survival of up to 78.9% out of all types of pineal tumors [9].

Psychiatric presentations are rare and atypical for pineal germinomas [3]. These presentations are found to be attributed to involvement of deep grey structures, e.g., thalamus and basal ganglia, infiltrative nature of tumor, or endocrine dysfunction [3]. Our patient had behavioral changes, including reduction in the amount of speech, hypersomnia, and psychomotor slowing. His pineal germinoma involved bilateral thalami, and this might attribute to his behavioral and psychiatric presentations. The thalamus is known to be responsible for higher nervous functions such as language, cognition, memory, and intelligence, whereas the diffuse thalamo-cortical projections are associated with maintenance mechanisms of consciousness such as sleep and wakefulness [10].

Pineal germinomas typically demonstrate increased attenuation relative to the grey matter on computer tomography scan, and a draped configuration with respect to the posterior third ventricle [8]. They are typically isointense or hyperintense to grey matter on T1- and T2-weighted MRI, with cystic and necrotic changes seen in larger masses [8]. MRI of the entire neuroaxis and lumbar puncture are recommended to assess for seeding in CSF and drop metastases [8].

Parinaud’s syndrome is a neurological syndrome that is alternatively known as sylvian aqueduct syndrome, dorsal midbrain syndrome, pretectal syndrome, and Koerber-Salus-Elschnig syndrome [11]. Parinaud’s syndrome results from dorsal midbrain lesions, e.g., tumors, hemorrhage, infarction, obstructive hydrocephalus, multiple sclerosis, inflammation, encephalitis, trauma, arteriovenous malformations, and tonic-clonic seizures [11]. The classical signs of Parinaud’s syndrome include convergence-retraction nystagmus, upgaze palsy, light near dissociation, and lid retraction [11].

Isolated findings of nystagmus may be challenging in localizing the lesion. However, Parinaud’s syndrome provides high localizing value of lesions. Convergence-retraction nystagmus is caused by damage to the midbrain supranuclear fibers, which have inhibitory effects on midbrain convergence or divergence neurons [11]. Limitation in conjugate upgaze occurs due to involvement of vertical gaze centers, i.e., rostral interstitial nucleus of medial longitudinal fasciculus and interstitial nucleus of Cajal, which are in proximity to the superior colliculus [11]. On the other hand, downgaze palsy occurs in reverse Parinaud’s syndrome, which is very rare, because downgaze is mediated by the medial portion of the rostral interstitial nucleus of medial longitudinal fasciculus [11].

One of the features of Parinaud’s syndrome is light-near dissociation, characterized by the dissociation between the pupillary light and near reflexes. It is due to damage of the pupillary light reflex fibers while sparing near reflex fibers [11]. Pupillary light reflex fibers synapse at the pretectal nucleus and then pass through the posterior commissure to the ipsilateral and contralateral Edinger-Westphal nucleus, making them susceptible to compression by mass lesions [11]. However, near reflex fibers are spared due to their more ventral location [11].

Lid retraction in the primary position, which is also known as the Collier's sign, may also occur in Parinaud’s syndrome [11]. It is caused by damage to the levator inhibitory fibers at the posterior commissure [11].

Pineal germinomas are treatable with radiation therapy alone or combined with neoadjuvant chemotherapy [12]. Radiotherapy may be directed to the whole ventricle or craniospinal area, with or without focal tumor irradiation [12]. The combination of neoadjuvant chemotherapy and radiotherapy has shown favorable outcomes in terms of tumor shrinkage rate and relapse-free survival rate [6,7].

## Conclusions

This case illustrates an uncommon combination of subacute behavioral changes with Parinaud’s syndrome and neurological deficits as the clinical presentation of a pineal germinoma. We highlight the rarity of such presentation to enhance the awareness of its potentially life-threatening risk, the importance of thorough history taking throughout the course of symptoms, the need of holistic physical examination particularly in neurological and ophthalmic examinations, and the role of multidisciplinary teamwork in the management of pineal germinomas to ensure a timely diagnosis and enhance comprehensive patient care.
